# Coexistence of virome-encoded health-associated genes and pathogenic genes in global habitats

**DOI:** 10.1128/aem.01501-25

**Published:** 2025-11-24

**Authors:** Min Qian, Dong Zhu, Ke-yu Yao, Shu-yue Liu, Meng-ke Li, Mao Ye, Yong-guan Zhu

**Affiliations:** 1 State Key laboratory of Soil and Sustainable Agriculture, Institute of Soil Science, Chinese Academy of Sciences 74586 , Nanjing, China; 2 National Engineering Laboratory of Soil Nutrients Management, Pollution Control and Remediation Technologies, Institute of Soil Science, Chinese Academy of Sciences, Nanjing, China; 3 University of Chinese Academy of Sciences, Nanjing, China; 4 Key Laboratory of Urban Environment and Health, Ningbo Observation and Research Station, Institute of Urban Environment, Chinese Academy of Sciences 85406 , Xiamen, People’s Republic of China; 5 Zhejiang Key Laboratory of Urban Environmental Processes and Pollution Control, CAS Haixi Industrial Technology Innovation Center in Beilun, Ningbo, People’s Republic of China; 6 State Key Laboratory of Urban and Regional Ecology, Research Centre for Eco-Environmental Sciences, Chinese Academy of Sciences 26442 , Beijing, China; Centers for Disease Control and Prevention, Atlanta, Georgia, USA

**Keywords:** virus, health-associated genes, global distribution, human disease, coexistence

## Abstract

Viral remnants constitute approximately 8% of the human genome, reflecting extensive historical gene exchange between viruses and their hosts. Some viral genomes harbor genes acquired through horizontal gene transfer that are associated with potential benefits to human health, alongside genes associated with pathogenicity. However, their global distribution, functional characteristics, and coexistence patterns remain poorly understood. Here, using the Integrated Microbial Genomes and Virome (IMG/VR v4) database, we identified 4,556 viruses carrying gene segments associated with human health across eight habitat types spanning 13 regions and 76 countries worldwide. Among viruses with identifiable hosts, those distributed in humans (478) accounted for the highest proportion. The viral genes associated with human health included *BCO1* (beta-carotene oxygenase 1), *bioB* (biotin synthase), *COQ2* (4-hydroxybenzoate polyprenyltransferase), *GPX1* (glutathione peroxidase 1), *GSTs* (glutathione transferases), *GSTT1* (glutathione S-transferase theta 1), *GULO* (L-gulonolactone oxidase), and *menA* (1,4-dihydroxy-2-naphthoate polyprenyltransferase). These genes not only associate with human health but also function as auxiliary metabolic genes in viral genomes. Notably, four pathogenic genes were found in viral sequences carrying health-associated genes, with potential for transcription and expression, indicating functional interactions. Experimental transduction of the viral *bioB* gene into *Escherichia coli* altered the expression of host pathogenic genes *GCH1* (GTP cyclohydrolase IA) and *UGDH* (UDP-glucose 6-dehydrogenase), supporting potential cross-regulatory interactions. Overall, this study incorporates health-associated genes into viral genomics, highlighting their coexistence with pathogenic genes, and provides new insights into virus-host coevolution and potential biotechnological applications.

**IMPORTANCE:**

Viruses are the most abundant biological entities on Earth and key drivers of microbial evolution through horizontal gene transfer. While often studied for their pathogenic effects, viruses can also carry genes that influence host metabolism and health. Genes associated with human health have been identified in viral genomes, yet their global distribution, functions, and coexistence with pathogenic genes remain largely unexplored. This study integrates datasets of health-associated genes into viral genomic analyses, revealing for the first time the coexistence of viral health-associated genes with those linked to pathogenicity. This dual genetic potential is observed across diverse habitats, highlighting viruses as multifaceted reservoirs of both beneficial and harmful genes. The study findings advance understanding of viral functional diversity and open new avenues for exploring viral roles in microbial ecology, biotechnology, and human health.

## INTRODUCTION

Viral remnants make up approximately 8% of the human genome, including sequences that encode genes potentially beneficial to human health as well as those associated with pathogenicity, underscoring the complex and long-standing relationship between viruses and their human hosts (1–5). Viruses have interacted with humans for over 300,000 years, influencing host adaptation, evolution, and disease propagation (2, 6, 7). Throughout history, virus-induced diseases have caused major public health crises, ranging from the 20th-century Spanish influenza (8) to HIV/AIDS in the 21st century (9) and, most recently, the COVID-19 epidemic (10). These events underscore the pathogenicity of viruses, primarily driven by virulence genes that disrupt host cell structures and functions, leading to various diseases (11–14).

Despite their pathogenic potential, viruses have also evolved genes that benefit human hosts through millennia of host-virus interactions (15, 16). Some viruses carry genes associated with potential health benefits, acquired through horizontal gene transfer (HGT) from host cells, forming auxiliary metabolic genes (AMGs) (16–21). These AMGs enhance viral adaptation to diverse environments and can positively influence host metabolism during infection (21–24). For example, the gene *psbA* (photosystem II P680 reaction center D1 protein) serves as a biomarker for classifying oceanic and freshwater environments and has potential applications in source tracking (24, 25). Similarly, genes *arsC* (arsenate reductase) and *arsM* (arsenite methyltransferase) in soil lysogenic viruses encode arsenic metabolism, influencing bacterial responses to environmental arsenic exposure (26). In marine environments, viral AMGs such as *glnK* (nitrogen regulatory protein P-II 2), *norB* (nitric oxide reductase subunit B), *nirK* (nitrite reductase), and *nirA* (ferredoxin-nitrite reductase) contribute to the nitrogen cycle (23). In nutrient-scarce environments like the South Pole, the viral gene *cl* enhances host survival by suppressing the expression of *pckA* (phosphoenolpyruvate carboxykinase) in the host, conferring selective advantages (27, 28). These findings highlight the role of AMGs in supporting host metabolism and environmental adaptation.

While the ecological roles of viral AMGs are increasingly recognized, significant knowledge gaps remain regarding virus-encoded proteins that are associated with human health or pathogenicity. The occurrence, functions, and geographic distribution of these health-associated genes in viruses remain incompletely characterized (29, 30). Health-associated genes are viral genes that are directly or indirectly linked to processes beneficial to human health, such as anticancer activity and vitamin synthesis, in contrast to pathogenic genes, which contribute to disease. Moreover, although viral genes that contribute to both human health and pathogenicity have been identified (13, 14, 27, 28), the mechanisms that allow these seemingly opposing functions to coexist remain poorly understood. Viruses may balance the expression of these genes through sophisticated regulatory networks, including transcriptional control and epigenetic modifications, to optimize survival and transmission across diverse hosts and environmental conditions (31–33).

**Fig 1:**
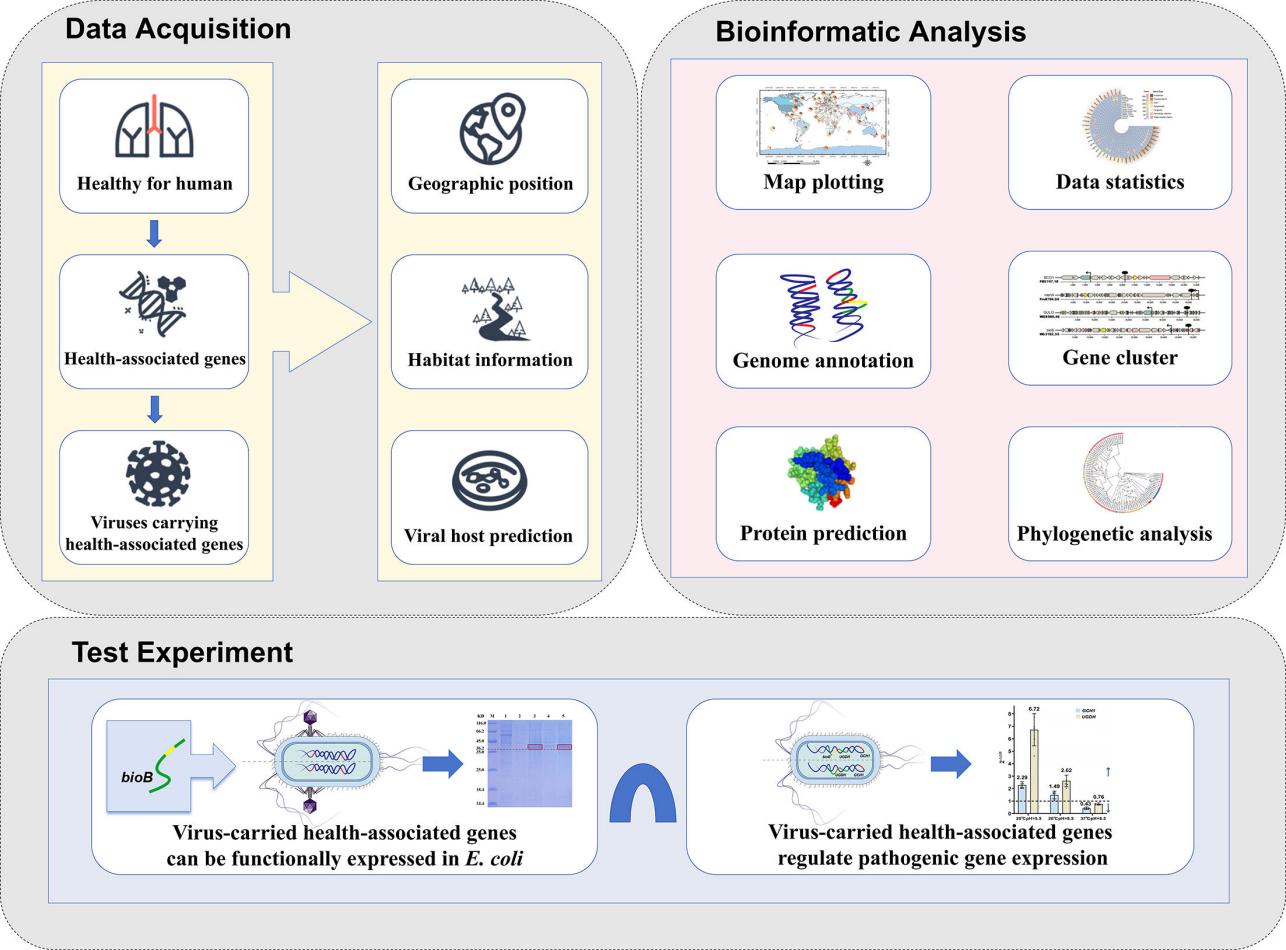
Research procedures.

To address these gaps, this study analyzed four representative types of health-associated genes, including anticancer, vitamin synthesis, antioxidant, and longevity genes, identified through a literature search (Table S1). Using the global public database Integrated Microbial Genomes and Virome (IMG/VR v4), the present study investigated the geological distribution and habitat characteristics of viruses carrying these gene sequences worldwide (Tables S2 and S3). Additionally, this research examined host-virus relationships and phylogenetic patterns (Tables S3 and S4) and explored the mechanisms balancing human health and pathogenic functions in viruses across different environments. By introducing health-associated genes into *Escherichia coli* to simulate phage-mediated transduction of the AMG *bioB* (biotin synthase), this study demonstrated that these genes significantly influence the differential expression of pathogenic genes *GCH1* (GTP cyclohydrolase IA) and *UGDH* (UDP-glucose 6-dehydrogenase) in the host (Table S6). These findings confirm that viruses can compensate for health-associated gene functions in hosts during HGT (Fig. 1). This study provides novel insights into the distribution, functions, and potential applications of viral genes involved in human health, advancing the understanding of host-virus dynamics in applied and environmental microbiology.

## RESULTS

### Extensive global distribution of viruses carrying health-associated genes

**Fig 2:**
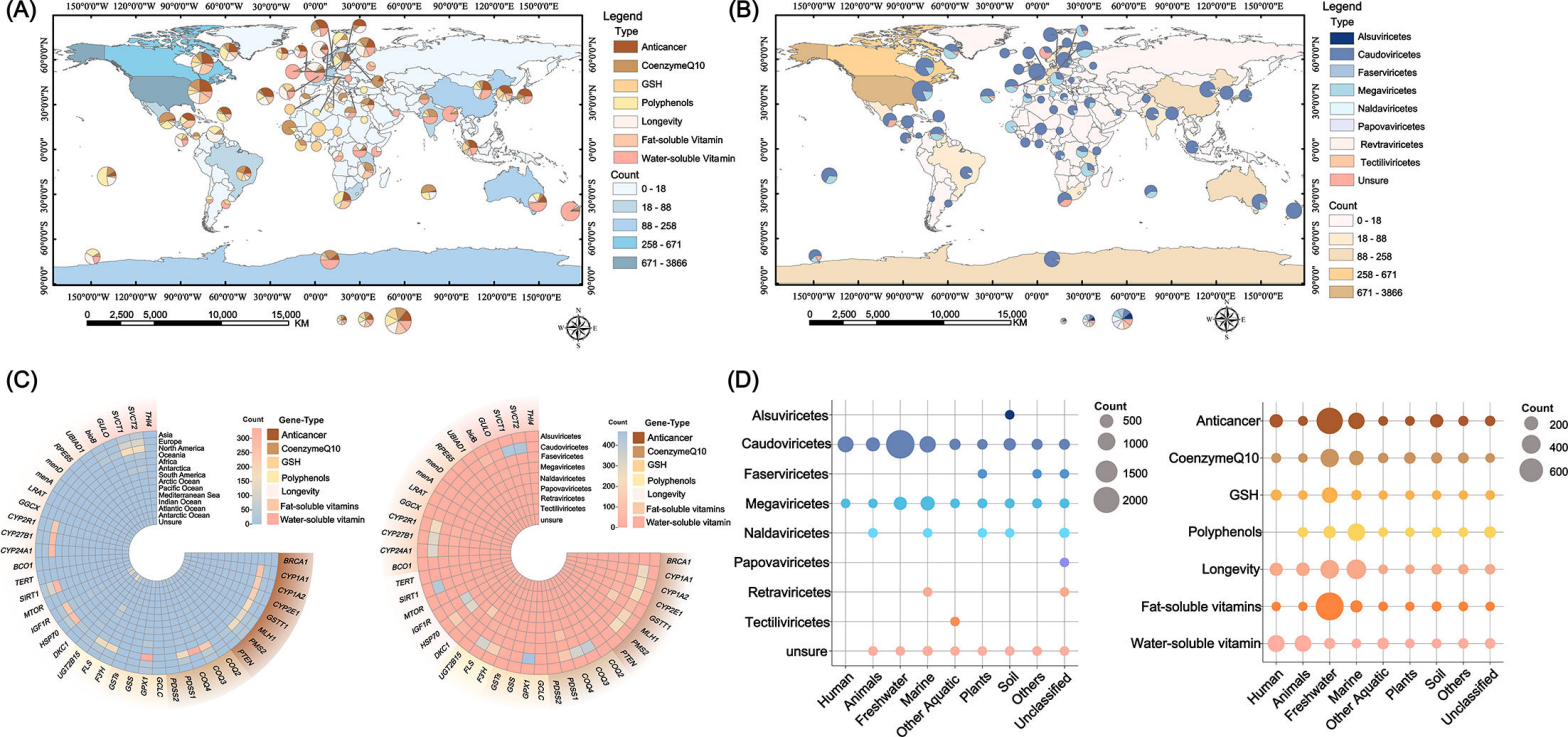
Global distribution of viruses carrying health-associated genes. (**A**) Global distribution of viruses carries different types of health-associated genes. Different regional colors represent the abundance range of viruses carrying health-associated genes. The radius of the pie chart represents an abundance of viruses carrying health-associated genes. The pie chart represents the proportions of viruses carrying different types of health-associated genes in a region. (**B**) Global distribution of different types of viruses includes health-associated genes. Different regional colors represent the abundance range of viruses carrying health-associated genes. The radius of the pie chart represents an abundance of viruses carrying health-associated genes. The pie chart represents the proportions of different types of viruses carrying health-associated genes in the region. The maps were created using ArcGIS 10.8, based on data from Natural Earth. (**C**) Heat map shows the quantitative relationship between viruses carrying health-associated genes and virus classes. (**D**) Bubble diagrams show the quantitative relationship between eight types of habitats and virus types, and bubble diagrams of the quantitative relationship between habitats and gene functions.

Fifty-five representative health-associated genes were identified, encompassing functions related to disease prevention and physiological resilience, including anticancer activity, vitamin biosynthesis, antioxidant production, and longevity support (34–41). These genes were screened across 7,159 uncultivated viral genomes (UVIGs), of which 6,583 contained geolocation metadata and 6,829 were linked to specific habitats. Viruses harboring health-associated genes were detected in 13 global regions spanning 76 countries. The highest numbers were observed in the USA (3,866), followed by Canada (671) and the UK (258) (Fig. 2A; Table S3). These viruses were classified into eight major viral taxa: *Alsuviricetes*, *Caudoviricetes*, *Faserviricetes*, *Megaviricetes*, *Naldaviricetes*, *Papovaviricetes*, *Revtraviricetes*, and *Tectiliviricetes*. Tailed bacteriophages (*Caudoviricetes*) encoded the largest number of health-associated genes (5,531), followed by giant viruses (*Megaviricetes*, 1,262) (Fig. 2B; Table S3). Among the 40 distinct health-associated genes identified, *HSP70* (heat shock 70 kDa protein) was the most frequently encoded (472 occurrences), followed by *GPX1* (glutathione peroxidase 1; 468) and *SVCT2* (solute carrier family 23 member 2; 448) (Fig. 2C; Table S3). The viruses encoding these genes were associated with diverse environments, grouped into eight categories: humans, animals, freshwater, marine, other aquatic, soil, plants, and others. The highest numbers were found in freshwater (2,758), followed by ocean water (1,511) and human-associated samples (769).

Functionally, these genes were categorized into seven groups: anticancer, coenzyme Q10, glutathione synthesis, polyphenol, longevity, fat-soluble vitamin, and water-soluble vitamin. Among these, anticancer-related genes were the most prevalent (1,540 occurrences), followed by genes associated with longevity (1,373) and water-soluble vitamin synthesis (1,006) (Fig. 2D; Table S3). Collectively, these findings reveal a broad global distribution of viruses encoding health-associated genes and provide theoretical support for understanding viral survival strategies and their potential roles in mediating beneficial host interactions.

### High host prediction rate in viruses carrying health-associated genes in human habitats

**Fig 3:**
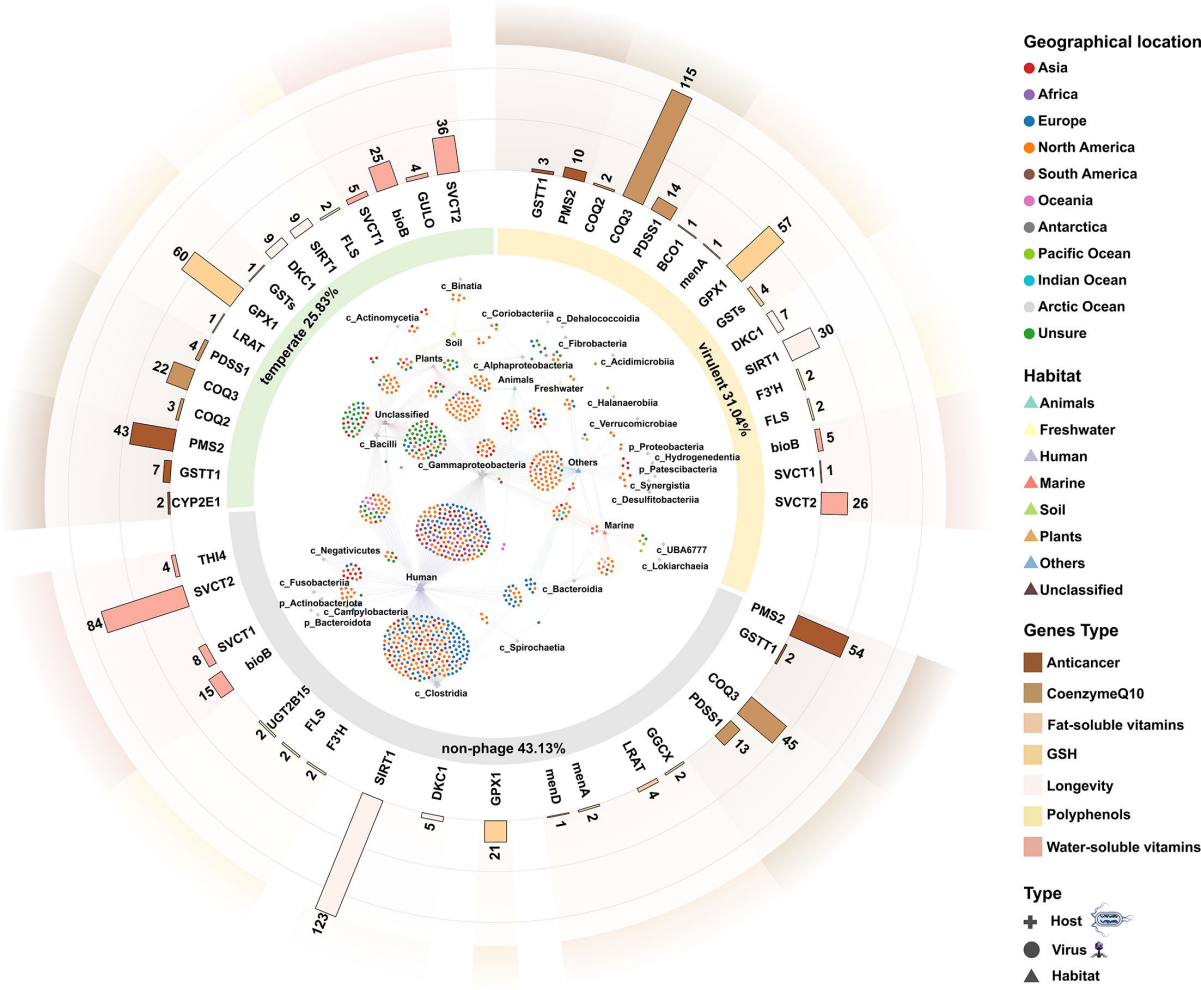
The host prediction diagram shows viruses carrying health-associated genes. Viruses were identified in equally large round charts. In the round charts, 11 colors represent global regional distributions, and the eight triangles in different colors represent the habitats. Cross-shaped markers represent predicted hosts. The larger marker represents the higher number of viruses belonging to this type of host or habitat. Specifically, each round point was related to the cross-shaped markers and triangles by two lines in different colors. The lines connecting the cross-shaped markers are silver, while the lines connecting with the triangles are the same color as the habitats. The green of the inner ring indicates lysogenic phages, orange shows virulent phages, and gray indicates non-phages. The colors of the outer ring represent classes of health-associated genes. The histogram corresponds to the inner ring, indicating the quantity of health-associated genes in the three types of viruses.

Host prediction analysis was performed on 4,556 viral sequences, of which 904 contained assignable host information, including 904 kingdom- and phylum-level annotations and 896 class-level assignments (Fig. 3; Table S2). Across these 904 viral sequences, 26 host types were predicted, with 22 classified at the class level and four unclassified beyond the phylum level. The dominant predicted hosts included c_*Gammaproteobacteria* (389 sequences), c_*Clostridia* (238 sequences), and c_*Bacilli* (128 sequences). Viral sequences were globally distributed across 11 regions: Asia, Africa, Europe, North America, South America, Oceania, Antarctica, Pacific Ocean, Indian Ocean, Arctic Ocean, and Unsure. The majority of sequences originated from North America (401 sequences), likely due to higher sampling density in that region.

Habitat analysis revealed that most host-associated viral sequences were linked to humans (478 sequences), followed by plants (64 sequences). Among 514 phage sequences, 280 were classified as virulent and 233 as temperate. Viruses carrying human health-associated AMGs were detected in both groups, with temperate phages harboring 22.3% more such genes per viral genome on average than virulent phages. Overall, phages carrying health-associated AMGs accounted for 4% of all phage sequences, whereas non-phage viruses carrying similar genes represented only 1% of total non-phage sequences.

### Virus-encoded health-associated genes exhibit the potential for transcription and translation

**Fig 4:**
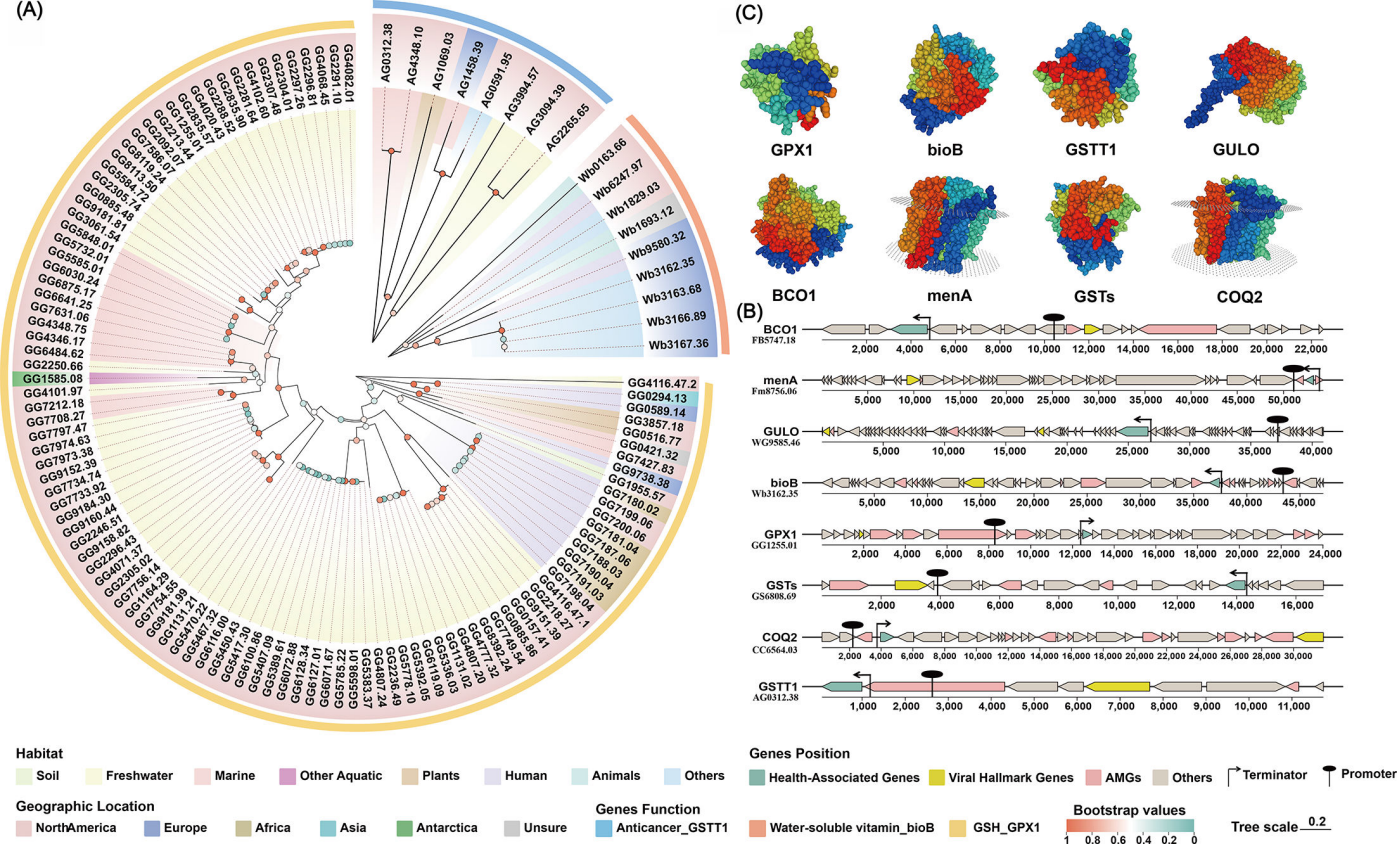
Phylogenetic tree of *GPX1*, *GSTT1*, and *bioB*; distribution in viral sequences, transcription, and translation of health-associated AMGs. (**A**) Phylogenetic evolutionary relations of *GPX1*, *GSTT1*, and *bioB* are in different viral sequences. On the outer ring, *GPX1*, *GSTT1*, and *bioB* are expressed in yellow, blue, and red, respectively. (**B**) Relative locations are shown among eight typical health-associated AMG clusters, promoters, and terminators. In the AMG clusters, health-associated genes are expressed in green, virus marker genes are expressed in yellow, other AMGs are expressed in red, and non-annotated genes are expressed in gray. (**C**) Four-level protein structures are predicted by health-associated AMGs.

Through DRAM-V and VIBRANT prediction tools (42, 43), *BCO1* (beta-carotene oxygenase 1), *bioB*, *COQ2* (4-hydroxybenzoate polyprenyltransferase), *GPX1*, *GSTs* (glutathione transferases), *GSTT1* (glutathione S-transferase theta 1), *GULO* (L-gulonolactone oxidase), and *menA* (1,4-dihydroxy-2-naphthoate polyprenyltransferase) were identified as virus-encoded health-associated AMGs (Table S5). In total, 133 viral sequences with high confidence carried both health-associated AMGs and virus marker genes. Phylogenetic and functional analyses revealed evolutionary relationships among *GPX1*, *GSTT1*, and *bioB* across different viral sequences (Fig. 4A; Table S4). Within the same geographic regions, such as North America, viruses carrying the *GPX1* gene from marine and freshwater habitats exhibited pairing distances of 0.3–0.4, indicating relatively close phylogenetic relationships. In contrast, viruses carrying *bioB* from human-associated samples in Europe displayed higher pairing distances (>0.9), reflecting stronger consanguinity ties (44–46). Notably, viruses carrying *GPX1* in humans from North America and Africa exhibited pairing distances near 0, indicative of exceptionally close evolutionary relationships (45–47). Promoter and terminator regions located immediately upstream and downstream of health-associated AMGs (*BCO1, bioB, COQ2, GPX1, GSTs, GSTT1, GULO*, and *menA*) were predicted, suggesting potential for transcription and translation into functional proteins (23, 48)(Fig. 4B). Structural models of these eight genes, generated using SWISS-MODEL, exhibited Global Model Quality Estimation (GMQE) indices of 0.56, 0.95, 0.95, 0.80, 0.61, 0.97, 0.94, and 0.92, indicating the capacity of viral sequences to encode proteins with complete structures and functional roles (44, 49, 50) (GMQE >0.5) (Fig. 4C).

### Pathogenic genes in viral genomes carrying health-associated genes

**Fig 5:**
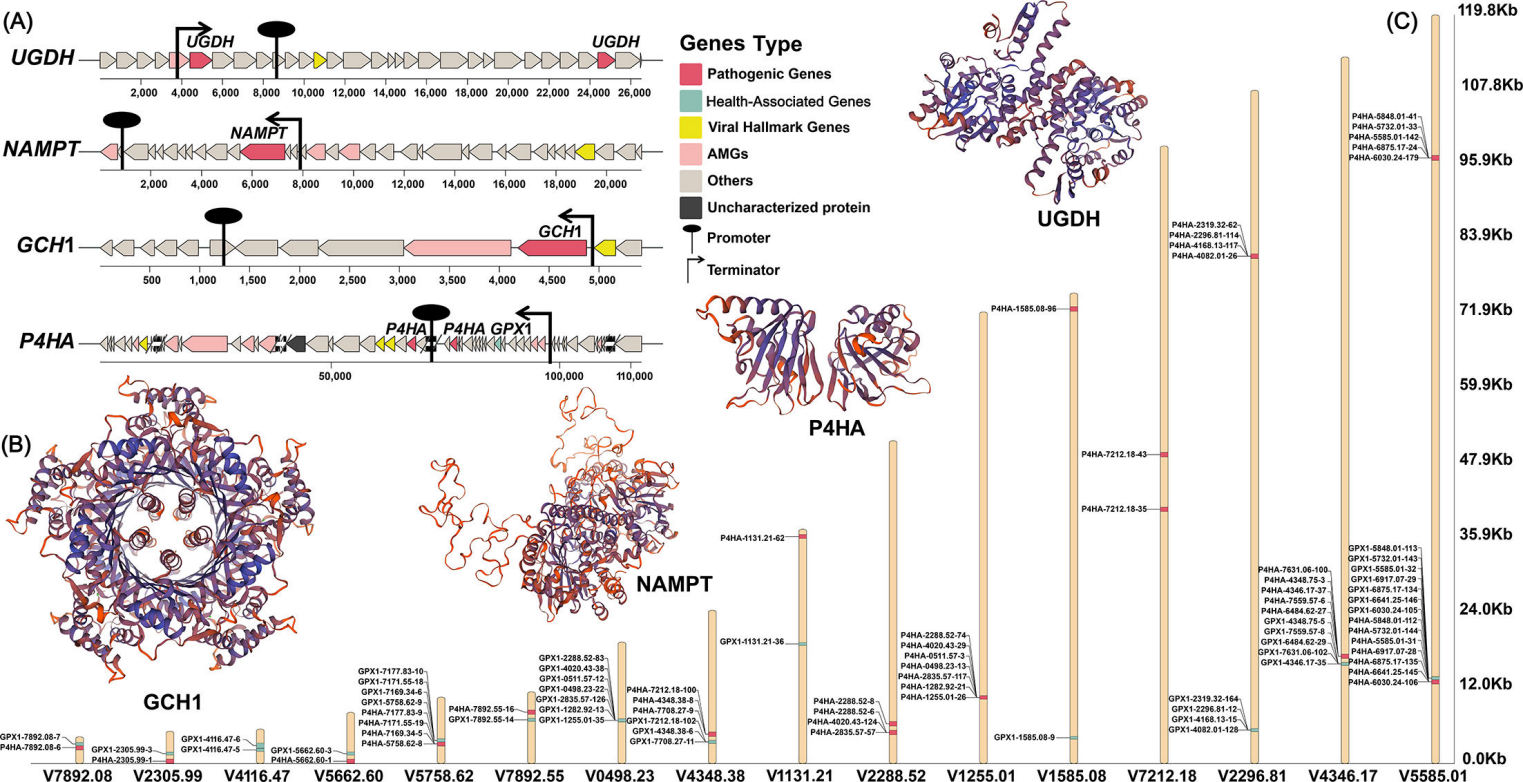
Expression potential of pathogenic genes and their coexistence with health-associated genes. (**A**) Pathogenic gene clusters in viruses are shown carrying health-associated genes. Pathogenic genes are in red, health-associated genes are in green, viral marker genes are in dark red, other AMGs are in blue, non-annotated genes are in yellow, and annotated genes whose functions have not been recognized are in gray. (**B**) Four-level protein structures are predicted by pathogenic genes. (**C**) Coexistence between *P4HA* and *GPX1* is in the viral sequences.

Four types of pathogenic genes (*GCH1*, *NAMPT* [nicotinamide phosphoribosyltransferase], *UGDH,* and *P4HA* [prolyl 4-hydroxylase]) were recognized in viral sequences carrying health-associated genes. These pathogenic genes were found to be transcriptionally active (Fig. 5A). Specifically, *GCH1* mutations can lead to tetrahydrobiopterin deficiency, resulting in phenylketonuria (37, 48, 51); elevated *NAMPT* expression may enhance cancer cell growth and survival, contributing to tumor development (52, 53); *UGDH* dysfunction can disrupt extracellular matrix composition, affecting tissue structure and function, with links to cancer and fibrosis (54); and increased *P4HA* expression may facilitate tumor progression and metastasis (55). Despite evidence of transcriptional activity, the GMQE values of the four types of pathogenic genes were 0.63, 0.53, 0.59, and 0.67 (Fig. 5B), which were generally lower than those of health-associated genes, suggesting a reduced likelihood of translation compared with health-associated genes (44, 49, 50). Notably, pathogenic and health-associated genes frequently co-occurred within viral genomes, typically separated by distances of ~1–2 kb (Fig. 5C), indicating a close genomic association.

### Balancing effects of environmentally mediated regulation of exogenous health-associated genes on the expression of pathogenic genes

**Fig 6:**
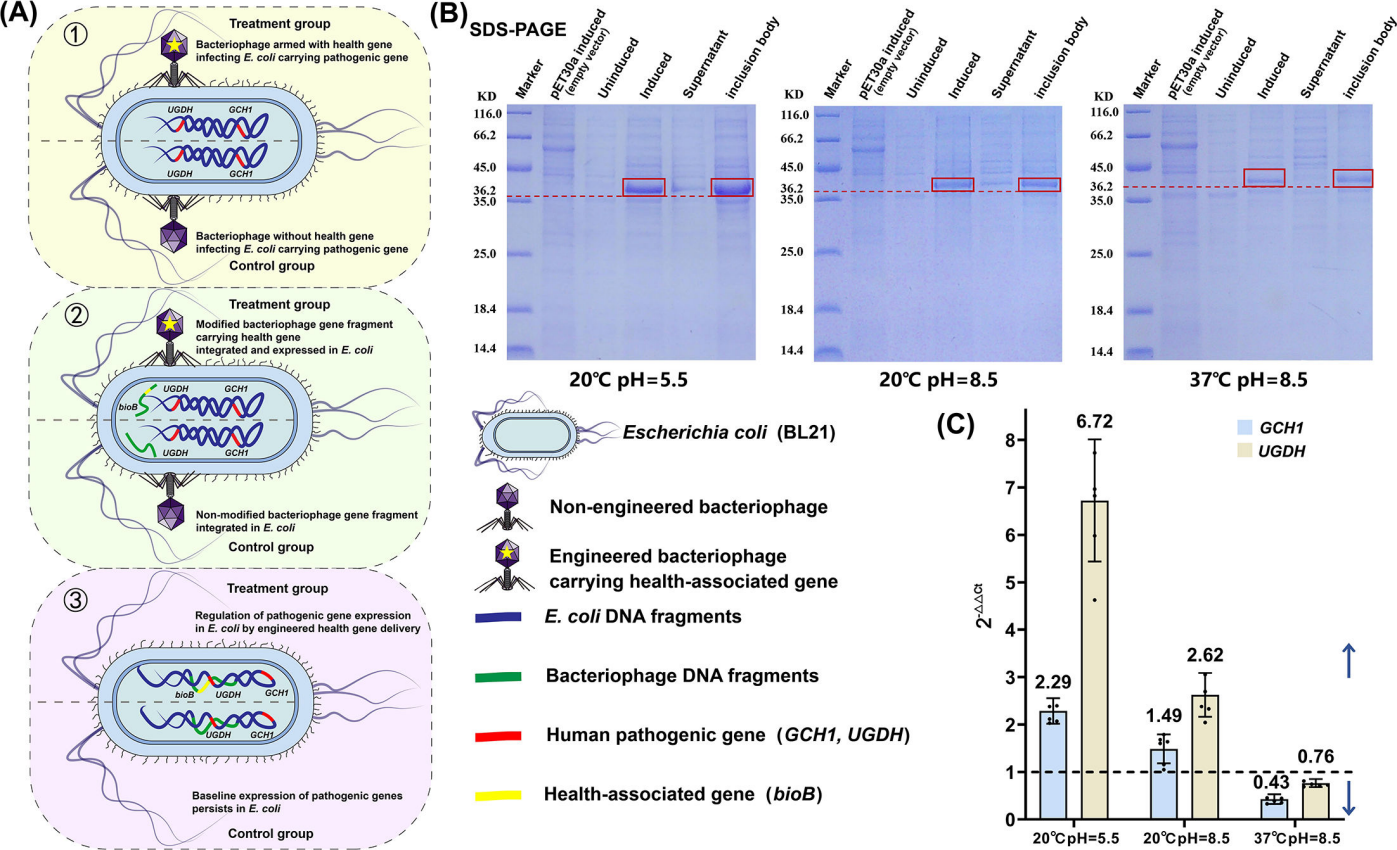
The functional expression of health-associated genes carried by viruses and the regulation of the expression of endogenous prophage pathogenic genes. (**A**) Schematic diagram of the experimental group and control group for simulating phage infection of *Escherichia coli* BL21. The annotations ①, ②, and ③ represent three steps (adsorption, injection, and integration) in transferring the *bioB* gene into *Escherichia coli*. Each colored section has treatment groups above and control groups below. Detailed information is given via figure captions and far-right annotations; (**B**) functional expression bands of the health-associated gene *bioB* on SDS-PAGE under different conditions; (**C**) simulation of the regulation of the expression levels of the pathogenic genes by the health-associated gene *bioB* under different natural environments. The bar chart, generated from a qPCR experiment with six replicate control sets, shows the fold change in relative expression levels of the target genes *GCH1* and *UGDH* in *Escherichia coli* transduced with the *bioB* gene compared to the control group, with standard deviations indicated.

The health-associated *bioB* gene was introduced via plasmid transduction into *E. coli* B21, which was previously confirmed through sequencing to naturally harbor the prophage-derived pathogenic genes *GCH1* and *UGDH* (Fig. 6A; Table S6). To assess protein expression under physiologically relevant conditions, recombinant cultures were subjected to various environmental regimes, including temperatures of 20°C, simulating ambient conditions, and 37°C, mimicking the human intestinal environment (56). The pH was varied across 5.5, reflective of urinary conditions (57), and 8.5, corresponding to a pathological colonic setting (58). Successful heterologous expression of *bioB* was confirmed by SDS-PAGE and Western blot analyses, with the most prominent bands observed in the supernatant and inclusion body fractions under 20°C/pH 5.5, 20°C/pH 8.5, and 37°C/pH 8.5 conditions (Fig. 6B; Fig. S1).

The transcriptional impact of *bioB* expression on the pathogenic genes was subsequently evaluated using real-time quantitative PCR with *gapA* as an internal reference. A control group consisting of the same *E. coli* strain transformed with an empty vector was included in all experimental conditions for normalization. Under acidic and ambient temperature conditions of 20°C and pH 5.5, the expression levels of *GCH1* and *UGDH* were found to be up-regulated to 2.3-fold and 6.7-fold, respectively, relative to the empty vector control. When the pH was elevated to 8.5 while maintaining the temperature at 20°C, a moderate up-regulation to 1.5-fold for *GCH1* and 2.6-fold for *UGDH* was still observed. In contrast, under the human gut-mimicking condition of 37°C and pH 8.5, a notable down-regulation was recorded, with expression levels reduced to 0.8-fold for *GCH1* and 0.4-fold for *UGDH* (Fig. 6C; Table S6). These findings collectively indicate that the functional expression of the health-associated *bioB* gene is associated with significant and environmentally modulated changes in the expression of pathogenic genes, with a particularly suppressive effect observed under conditions simulating the human intestinal environment.

## DISCUSSION

### Potential impact of database completeness and sequence quality on ecological inferences

Large-scale metagenomic analyses often face a trade-off between maximizing data completeness and maintaining sequence quality. The IMG/VR database, by including fragmented and low-quality (LQ) viral genomes, provides a more comprehensive census of viral sequence space, which is valuable for assessing macroecological trends. In the analysis of habitat associations and geographic distribution, these LQ genomes were retained to minimize sampling bias against underrepresented viral groups (Table S2). It is established that LQ genomes may contain host-derived contamination; however, for analyzing relative distribution patterns across environments, this introduces a conservative, non-systematic error. The consistent and statistically significant patterns observed across diverse, independently sampled habitats support that the inferred habitat and geographic distributions are robust and unlikely to be generated solely by host contamination. Nevertheless, it is emphasized that all subsequent in-depth analyses of gene function and evolution were rigorously restricted to high-quality viral genomes to ensure the validity of the predictions regarding transcription and translation.

### Global habitat distribution and phylogenetic development of health-associated genes carried in viruses

Globally, viruses carrying health-associated gene segments were distributed across diverse habitats, with freshwater environments showing the highest account (38.5%)(Fig. 2; Table S3). This predominance likely reflects the rich organic matter, high microbial diversity, and dense host populations in freshwater systems, which provide favorable ecological niches for viral replication and persistence (59, 60). Geographically, the majority of these viruses were detected in the Americas (54.0%), indicating potential biases linked to sampling density, as well as possible biogeographic preferences associated with habitat type and host range diversity. Interestingly, many of these viral genomes contained genes associated with anticancer functions, suggesting a possible evolutionary strategy favoring host survival and adaptation within dynamic ecosystems (61, 62). Alternatively, anthropogenic pressures, such as pollution and elevated cancer incidence associated with environmental stress, may have indirectly selected for viruses harboring such health-associated genes as a response to altered ecological conditions (33, 63). Although the Americas exhibited the highest number of viral sequences carrying health-associated genes, the average number of such genes per viral genome was greatest in Honduras (3.33), followed by South Africa (2.62), and lower in the broader Americas (1.50). This pattern may reflect the high biodiversity and ecological complexity of Honduras, which could enhance opportunities for viral gene acquisition and retention (64). Similarly, dense human populations and limited public health infrastructure in parts of South Africa may promote viral adaptation through the selection and maintenance of health-associated genes (65, 66). These findings underscore the importance of future research in ecologically diverse but underexplored regions to establish more comprehensive and objective global patterns in the distribution and evolution of viral health-associated genes.

Phylogenetic analyses revealed that viruses carrying identical health-associated genes within the same habitat displayed high sequence similarity, consistent with descent from common ancestral genes. These genes likely play pivotal roles in viral adaptation and survival, explaining their strong conservation across similar ecological contexts (67). Habitat type and geographic position jointly influenced the evolutionary relatedness of these viruses. For instance, in North America, viruses carrying *GPX1* exhibited close genetic relatedness between marine and freshwater environments (pairwise distance: 0.3–0.4), suggesting limited habitat separation and consistent selective pressures. Conversely, in Europe, viruses carrying *bioB* displayed marked genetic divergence between human-associated and environmental habitats (pairwise distance >0.9), reflecting strong habitat isolation and niche-specific evolution. Notably, viruses carrying *GPX1* in human-associated samples from North America and Africa exhibited nearly identical genomic features, implying historical human movement, convergent selective pressures, or similar ecological conditions across continents (68). Collectively, these findings highlight the profound influence of both geography and habitat on the evolutionary trajectories of viral genomes carrying health-associated genes.

### Mechanisms underlying trade-offs between phage-transduced health-associated gene and pathogenic gene expression in the host

In the test experiment, the virus-carried *bioB* gene was successfully transduced into *E. coli* BL21, demonstrating the integration of a health-associated AMG and its regulatory influence on host pathogenic genes (69, 70). Under host-simulated physiological conditions (37°C, pH 8.5), *bioB* activity exhibited an antagonistic relationship with the pathogenic genes *GCH1* and *UGDH*, indicating that up-regulation of health-associated genes can suppress the expression of bacterial pathogenic genes. This suppression may reduce the transmissibility of bacterial virulence. Conversely, under environmental stress conditions (20°C, pH 5.5), transient activation of pathogenic genes may enhance the transmission potential of virulence determinants.

The coexistence of pathogenic and health-associated genes may arise from cascade regulation (71) or co-acquisition via horizontal transfer (72). Over evolutionary timescales, viruses appear to finely regulate both pathogenic and health-associated genes to balance virulence and host advantage. Viruses carrying pathogenic genes infect hosts more efficiently, replicate within them, and thereby ensure transmission and survival. These genes enhance pathogenicity and help overcome host defenses, improving transmission efficiency (73). In contrast, carrying health-associated genes strengthens host adaptation and survival, which indirectly benefits viral persistence. Some phages integrate health-associated genes into host genomes after infection, further enhancing host environmental adaptation (74, 75). By carrying and regulating both pathogenic and health-associated genes, viruses achieve a dynamic equilibrium between exploiting host resources and supporting host survival, ensuring long-term persistence. This balance reflects the complex interplay of host-virus interactions, simultaneously promoting viral adaptation and modulating disease transmission (76–79). Understanding these mechanisms may offer novel strategies to suppress the expression of pathogenic genes while enhancing health-associated genes, potentially reducing risk and supporting human health (80, 81).

Genomic analyses reveal that eukaryote-to-virus gene transfer occurs over twice as frequently as the reverse (82), highlighting the potential for health-associated genes to enter viral genomes. Viruses thus act as vectors for both pathogenic and health-associated genes, facilitating their dissemination. However, whether these virally transported eukaryotic genes can integrate into the human genome and exert functional effects remains unresolved. Future studies should systematically screen human genomes for virus-derived sequences and assess their functional impacts in model systems to clarify the physiological and health implications of virus-mediated gene flow.

## MATERIALS AND METHODS

### Literature review and data review

To define the concept of “health-associated genes,” a comprehensive literature search was conducted to identify genes directly or indirectly linked to processes that benefit human health. Peer-reviewed articles published over the past 5 decades were retrieved from X-MOL (https://www.x-mol.com) and Web of Science (https://www.webofscience.com/wos) using keywords including “anticancer gene,” “vitamin synthesis gene,” “antioxidant gene,” and “longevity gene.” From the screening of 58 relevant studies, a non-redundant set of 55 genes was compiled. These genes, which collectively define the health-associated gene category in this study, were classified into four functional groups: 18 involved in vitamin synthesis (e.g., *bioB* [34] and *menA* [35]), 14 with antioxidant functions (e.g., *COQ2* [36, 37] and *GPX1* [38]), 14 linked to anticancer proteins (e.g., *GSTT1* [39]), and nine associated with longevity (e.g., *SIRT1* [NAD-dependent protein deacetylase sirtuin 1]) (40) and *IGF1R* (insulin-like growth factor 1 receptor) [41]) (Table S1).

### Acquisition and comparison of sequences

Amino acid and nucleotide sequences of relevant genes were downloaded from the KEGG database (83). Gene names, KO numbers, and function information were collected. The target amino acid sequences were compared through the IMG/VR v4 database (84), obtaining the global viral sequences encoding health-associated genes (E-value <0.0001). A total of 8,999 sequences that met the criteria (bit score >60, identity >20%) were screened, resulting in 4,556 non-redundant viral sequences and 548 high-quality sequences (85, 86). Global spatial visualization was carried out through ArcGIS 10.7.

### Viral genome scanning and homology search

Viral genomes were scanned by HMMER (v.3.1b2) (87). All viral protein sequences were compared against the HMM profiles of the ViPhOG database, which contains curated orthologous groups of viral proteins (86, 87). Matches with an E-value ≤1e^−5^ were considered significant, and only the highest-scoring hit for each sequence was retained to minimize redundancy and false positives.

### Gene annotation and cluster visualization

Functional annotation was performed using DRAM-V (v.1.2.0), which identifies viral marker genes and AMGs (42). In instances where the auxiliary score falls below 4, the M flag is assigned, while the A, V, or T flags are not assigned. This designation of a gene as a potential AMG is based on the specific numerical values assigned to each flag (42). Predictions were cross-validated with the vitality pipeline (v.1.2.0)(43). Annotation results were further compared against the KEGG database to confirm functional assignments. Genes with ambiguous functions were retained only if multiple databases gave consistent annotations.

### Promoter and terminator prediction

Regulatory elements were predicted using BPROM and FindTerm on the Softberry platform (http://www.softberry.com/) (88, 89). BPROM-predicted promoters were filtered to retain only those with linear discriminant function >2.75, while FindTerm predictions of terminators were ranked by confidence, with only the highest-confidence sites retained. Predicted promoters and terminators were manually inspected to ensure genomic context consistency with viral genome architecture (88, 89).

### Protein structure prediction

Protein tertiary and quaternary structures were predicted through the SWISS-MODEL server (https://swissmodel.expasy.org/) (90). Models were generated with default parameters, and only those with GMQE >0.5 were retained to ensure structural reliability. GMQE is a quality score provided by SWISS-MODEL, ranging from 0 to 1, which predicts the expected accuracy of the resulting protein structure based on target-template alignment and template quality; higher GMQE values indicate more reliable models (44, 49, 50). Gene annotation information, including viral marker genes and AMGs, was visualized and refined using Chiplot (https://www.chiplot.online/), enabling clear display of gene types and functional categories (91, 92). Gene clusters carrying health-associated AMGs were only selected for visualization if they also contained confirmed viral marker genes, ensuring that the displayed clusters represent bona fide viral genomic contexts.

### Phylogenetic analysis

High-quality amino acid sequences were aligned using ClustalW implemented in MEGA11 (93, 94). Phylogenetic trees were constructed using the neighbor-joining method, with bootstrap analysis based on 1,000 replicates to assess the robustness of the branching topology. Bootstrap values were calculated for all nodes and displayed on the final tree (45). The resulting trees were exported and refined using the Chiplot online platform, where branch labels and functional annotations of genes were added for visualization.

### Gene positional analysis

The genomic positions of pathogenic genes and health-associated genes within viral genomes were visualized using the “Gene Location Visualize” function in TBtools (95), allowing assessment of their spatial relationships.

### Bacterial strain and culture conditions

The *Escherichia coli* BL21 strain used in this study endogenously harbors the pathogenic genes *GCH1* and *UGDH*. This strain was transformed with the plasmid pET30a-*bioB*, which carries the health-associated gene *bioB*, to generate the experimental strain. Transformants were selected on Luria-Bertani agar plates containing 50 µg/mL kanamycin. For protein expression, cultures were grown under varying temperature conditions (20°C and 37°C) and pH gradients (5.5, 7.0, 8.5, and 9.0). Protein expression was induced with isopropyl β-d-1-thiogalactopyranoside at a final concentration of 0.2 mM (for 20°C cultures) or 0.5 mM (for 37°C cultures) when the OD_600_ reached 0.6–0.8.

### Protein expression analysis by SDS-PAGE and Western blot

Cells were harvested and disrupted by ultrasonication. The soluble (supernatant) and insoluble (inclusion body) fractions were collected and analyzed. Proteins were separated on 12% SDS-polyacrylamide gels and visualized using Coomassie Brilliant Blue staining. For Western blotting, proteins were transferred onto a polyvinylidene fluoride membrane, which was subsequently blocked and incubated with primary antibodies, followed by horseradish peroxidase-conjugated secondary antibodies. Protein bands were detected using an enhanced chemiluminescence detection system.

### Genetic confirmation and gene expression analysis by quantitative real-time PCR (qRT-PCR)

The presence of the endogenous *GCH1* and *UGDH* genes in the bacterial genome was confirmed by sequencing of PCR-amplified products (Table S6). Total RNA was extracted from bacterial cultures, reverse-transcribed into cDNA, and analyzed by qRT-PCR using a SYBR Green-based system. The *gapA* gene was used as an endogenous control for normalization. Relative gene expression levels were calculated using the 2^−ΔΔCt^ method (96). All experiments were performed in six independent replicates and included an empty vector control to ensure reliability.

## Data Availability

All gene sequences in this research were sourced from KEGG, while all viral sequences were obtained from the IMG/VR v4 database. The genes KEGG Orthology numbers and viral sequence UViGs used in this research are provided as Supplementary Tables in the Supplementary materials. All data are publicly accessible for download.

## SUPPLEMENTAL MATERIAL

Fig. S1 aem.01501-25-s0001.tif Protein identification and analysis by Western blot.

Supplemental tables aem.01501-25-s0002.xlsx Tables S1 to S6.

ASM does not own the copyrights to Supplemental Material that may be linked to, or accessed through, an article. The authors have granted ASM a non-exclusive, world-wide license to publish the Supplemental Material files. Please contact the corresponding author directly for reuse.
