# Supplementary figures and images for: Coexistence of virome-encoded health-associated genes and pathogenic genes in global habitats
Source: Appl Environ Microbiol. 2025 Nov 24;91(12):e01501-25. doi: 10.1128/aem.01501-25 (PMC12724203; doi:10.1128/aem.01501-25)

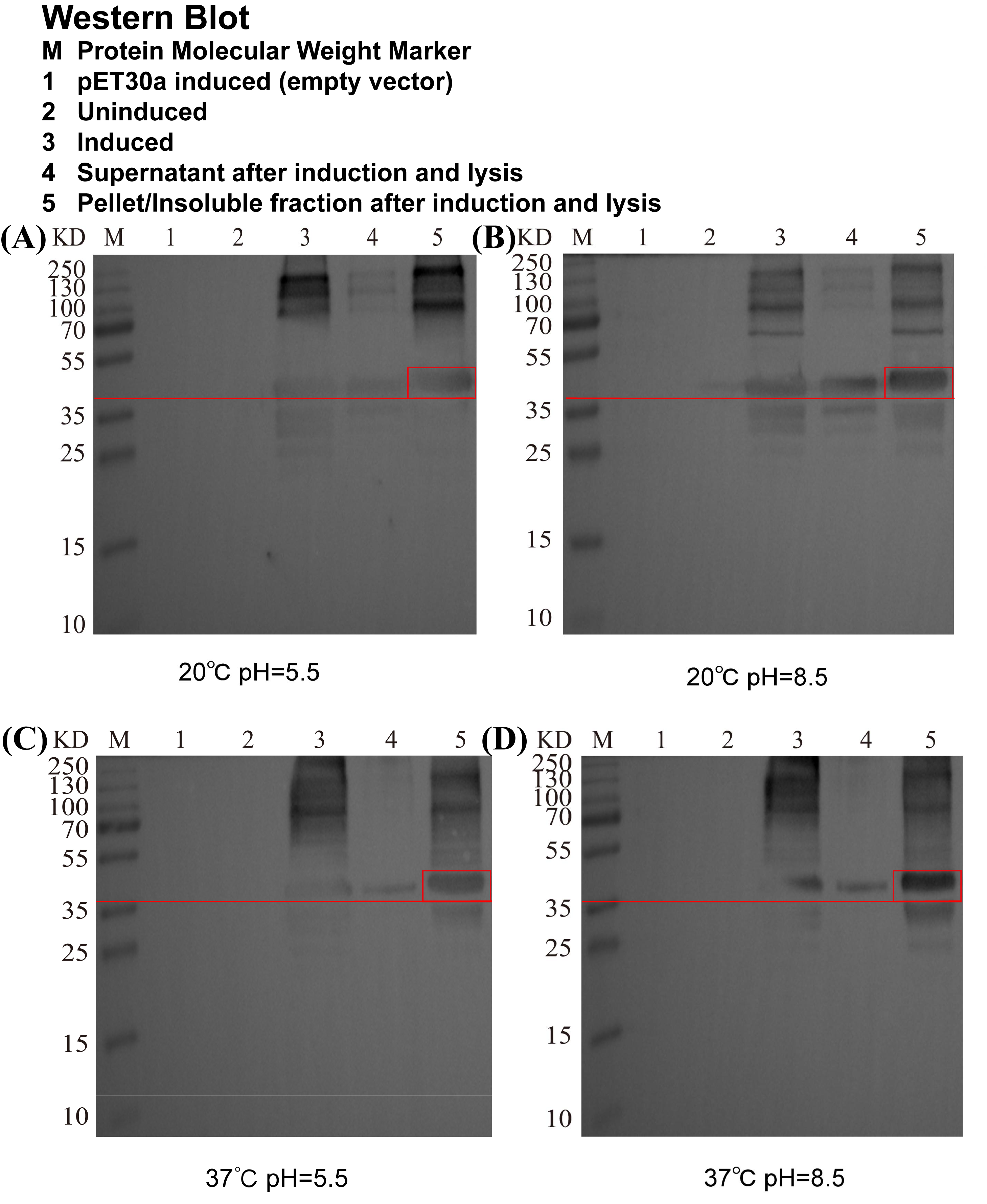

Supplement: Fig. S1 — Protein identification and analysis by Western blot. [file aem.01501-25-s0001.tif]
